# Hospitalizations for Major Cardiovascular Events in Patients Aged 75 Years or Older with Chronic Coronary Syndrome for the Whole Life Span

**DOI:** 10.3390/jcm15010207

**Published:** 2025-12-27

**Authors:** Lucas Barreiro Mesa, Martín Ruiz Ortiz, Josué López Baizán, Leticia Mateos de la Haba, Cristina Ogayar Luque, José Javier Sánchez Fernández, Elías Romo Peñas, Mónica Delgado Ortega, Ana Rodríguez Almodóvar, Fátima Esteban Martínez, Manuel Anguita Sánchez, Rafael González Manzanares, Juan Carlos Castillo Domínguez, José López Aguilera, Amador López Granados, Manuel Pan Álvarez-Ossorio, Dolores Mesa Rubio

**Affiliations:** 1 Cardiology Department, Hospital Universitario Reina Sofía, University of Córdoba, 14004 Córdoba, Spain; lucasbarme5@gmail.com (L.B.M.); josuelopezbaizan@gmail.com (J.L.B.); leticiamateos96@gmail.com (L.M.d.l.H.); cologayar@hotmail.com (C.O.L.); sancfern@hotmail.com (J.J.S.F.); eliasromo@gmail.com (E.R.P.); modeortega@gmail.com (M.D.O.); anarodriguezal@yahoo.es (A.R.A.); fatimaesteban@hotmail.com (F.E.M.); manuelanguita@secardiologia.es (M.A.S.); rafaelglezm@gmail.com (R.G.M.); jcastillod@medynet.com (J.C.C.D.); mircardjla@gmail.com (J.L.A.); amadorlg@gmail.com (A.L.G.); manuelpanalvarez@gmail.com (M.P.Á.-O.); loladoctora@gmail.com (D.M.R.); 2 Maimónides Biomedical Research Institute of Córdoba (IMIBIC), University of Córdoba, 14004 Córdoba, Spain; 3 Cardiovascular Diseases Biomedical Research Networking Centre (CIBERCV), Carlos III Health Institute, 28029 Madrid, Spain; 4 Faculty of Health Sciences, Isabel I International University of Castile, 09003 Burgos, Spain

**Keywords:** chronic coronary syndrome, elderly patients, prognosis, hospitalizations, major cardiovascular events

## Abstract

**Background/Objectives:** Limited information exists on the burden of major cardiovascular morbidity in elderly patients with chronic coronary syndrome (CCS). Our objective was to investigate the cumulative incidence of lifetime hospitalizations for major cardiovascular events (MCE) in patients aged 75 years or older with CCS and to identify clinical predictors of these events. **Methods:** All consecutive outpatients aged 75 years or older with CCS seen in two consultations at a tertiary hospital between 2000 and 2008 were included in a prospective study and followed until death. All MCEs requiring admission (hospitalizations for heart failure (HF), acute myocardial infarction, and stroke) were recorded, and the cumulative incidence of each event and the combination of all events was calculated, considering death without admission as a competing event. **Results:** A total of 414 patients were selected (mean age was 79 ± 4 years, 36% women). After a 22-year follow-up (median 7 years, p25–75 4–11), 198 patients (48%) experienced at least one MCE, the most common being hospitalization for HF (122 patients had 209 hospitalizations). The 10 and 20-year cumulative incidence was 41% (95% CI 36–46%) and 48% (43–53%) for any event. In multivariate analysis, independent predictors of hospitalization for MCE were hypertension (HR 1.58 [95% CI:1.15–2.18], *p* = 0.005), diabetes (HR 1.38 [1.03–1.85], *p* = 0.031), prior HF (HR 2.52 [1.59–4.01], *p* < 0.0005), and atrial fibrillation (HR:1.68 [1.13–2.50], *p* = 0.011). **Conclusions:** Nearly half of elderly patients with CCS were hospitalized for MCE during their lifetime. HF was the most common event. Several clinical variables could be useful to stratify the risk of events.

## 1. Introduction

The prevalence of chronic coronary syndrome (CCS) has increased in recent decades, primarily due to the advancement in the management of acute coronary syndrome, which has reduced short-term mortality rates [1,2,3]. Historically, the prognosis of CCS has received less attention in our environment, especially in the subgroup of elderly patients. Our group’s previous report indicated that the mortality rate among CCS patients was higher than that of the general Spanish population [4], a finding that is particularly relevant in light of the progressive demographic aging of our country, with an estimated population pyramid inversion by the year 2050 [5]. 

However, elderly patients are frequently underdiagnosed, inadequately treated, and scarcely represented in most clinical trials [6,7,8]. The complexity of older patients, who often have multiple comorbidities, complicates the development of appropriate prognostic models [7,8,9,10]. This issue is further exacerbated by the challenge of conducting prolonged follow-ups in this demographic, leading to a paucity of information for patients aged ≥75 years with CCS. Our group previously published an analysis on long-term mortality predictors in patients aged ≥75 years with CCS [11]. However, due to the aforementioned limitations, there are no studies in the literature analyzing the lifetime incidence of hospitalizations for major cardiovascular events (MCE). The present study aims to examine the prevalence of acute myocardial infarction (AMI), stroke, or heart failure (HF) in individuals aged 75 years and over with chronic kidney disease (CKD), as well as the associated risk factors. It is imperative that healthcare systems develop a comprehensive understanding of this information to facilitate the formulation of effective therapeutic strategies for these populations, which are growing in number. The objective of this study was twofold: first, to investigate the cumulative incidence of lifetime hospitalizations for MCE in patients aged ≥75 years with CCS; and second, to identify clinical predictors of these events.

## 2. Materials and Methods

### 2.1. Study Design and Patients

The CICCOR registry (Chronic Ischemic Cardiopathy in CORdoba) is an observational, prospective, single-centre cohort study aimed at investigating the prognosis of CCS [4,11,12,13]. From February 2000 to December 2008, all consecutive patients with CCS who attended two general cardiology consults at a tertiary hospital in our country were prospectively recruited and followed until their death. Given that our study aimed to analyse hospitalisations for major cardiovascular events throughout the whole life span, those patients who were still alive at the end of the follow-up period were excluded from the analysis. The same principle applied to patients lost to follow-up.

CCS was defined as one or more of the following: a history of acute coronary syndrome (unstable angina or AMI) or coronary revascularization at least 3 months prior to inclusion; a history of chest pain with ergometry or imaging consistent with ischemia; or coronary angiography showing >70% stenosis of an epicardial vessel, without severe valvular heart disease. Only patients who declined to participate in the study were not included in the study.

### 2.2. Data Collection and Event Definition

Demographic, clinical, complementary test, and treatment variables were collected at the initial visit. Acute coronary syndrome (unstable angina or AMI) was defined as a clinical diagnosis based on the presence of compatible symptoms, 12 lead electrocardiogram findings, and rise and fall of biomarkers reflecting cardiomyocyte necrosis, according to the clinical practice guidelines in effect at the time of inclusion [14,15,16]. Revascularization was reported as percutaneous or surgical if any of these procedures had been performed in the patient (some patients had both types of revascularization performed). Medication use reflects prescriptions at the baseline visit. Patients were managed at the discretion of their responsible cardiologists, according to the clinical practice guidelines in effect at the time [14,15,16]. Follow-up for all patients was updated as of 31 December 2024, through consultation of electronic medical records, contact with primary care physicians, or patient’s relatives in selected cases. All events were systematically and individually assigned by the researchers based on these data sources, and no events were extracted from coded databases, so changes in coding should not have affected the results.

Stroke was defined as hospital admission for a focal neurological deficit lasting more than 24 h, confirmed by a neurologist in the discharge report. AMI was considered as hospital admission for ischemic chest pain with elevation of biochemical markers of myocardial damage, according to the definition current at the time of admission. Hospitalization for HF was defined as a hospital stay of at least one night due to symptoms of dyspnoea or edema, associated with pulmonary crackles, elevated central venous pressure, interstitial or alveolar edema on chest X-ray, requiring treatment with intravenous diuretics or inotropes. Out-of-hospital events were not included in the analysis, but all short stays were captured, if they included at least one night in hospital. 

### 2.3. Statistical Analysis

The normality of quantitative data was assessed using the Kolmogorov–Smirnov test and presented as mean ± standard deviation or median [interquartile range, p25–p75], as appropriate. Qualitative variables are expressed as percentages. Student’s T-test was used for the comparison of quantitative variables, and the Chi-squared test for qualitative variables, with Fisher’s exact test when necessary.

The cumulative incidences of the combined event and its components were calculated using the Aalen–Johansen estimator, accounting for all-cause mortality as a competing event. Cause-specific Cox proportional hazards models were used for time-to-event analyses to isolate the association of the baseline parameters with the events. First, associations were assessed using univariable Cox models. Subsequently, multivariable models were fitted, initially incorporating all variables with *p* < 0.15 in the univariable analysis. A backward stepwise elimination method was applied to identify independent predictors. Multicollinearity was assessed using variance inflation factors with a threshold of <5. Events per variable were calculated in all final multivariable models to assess overfitting. Although we recognise that multivariate analysis involves constructing a complex mathematical model in which all statistically significant variables influence the outcome together, we decided to maintain the term “independent predictors of events” to refer to explicative variables included in the final multivariate models because of their widespread use in literature [1,11]. Results are presented as hazard ratio (HR) and 95% confidence intervals (CI 95%). The assumption of proportional hazards was checked by analyzing Schoenfeld residuals. To further confirm the robustness of our findings, we performed two sensitivity analysis: first, we used a more parsimonious/stricter variable selection strategy (*p* < 0.10 for retention instead of *p* < 0.15), and second, we repeated the analysis with a forward stepwise inclusion method. Values of *p* < 0.05 were considered significant. As an investigational proposal, a risk score was assembled according to the method described by Rassi et al. [17], and the performance in our series was reported. Each regression coefficient of variables independently associated with MCE hospitalizations in the final multivariate Cox model was divided by the smallest coefficient and rounded to the nearest integer. R statistical software version 4.4.3 (R Foundation for Statistical Computing, Vienna, Austria) was used for cumulative incidence analysis, and IBM SPSS version 25.0 (Chicago, IL, USA) for the remaining calculations.

## 3. Results

### 3.1. Sample Characteristics

Of 421 patients initially selected, 414 were known to have died by December 31 of 2024 (5 were lost to follow-up and 2 were still alive), constituting the study sample. 

The mean age was 79 ± 4 years, with 36% being women. Most patients presented with dyslipidemia (72.8%), almost a third were diabetic, slightly over 60% were hypertensive, and 24% reported angina symptoms at the first visit. Regarding the diagnostic criteria for CCS, 377 patients (91.3%) had a previous acute coronary syndrome, 108 (26.2%) had previous revascularization, and 22 (5.3%) had angina with evidence of ischemia. More than half of the patients showed an altered electrocardiogram at the first visit, and 12% had already been diagnosed with atrial fibrillation. The mean ejection fraction was 54 ± 15%. Antiplatelet agents were received by 86% of patients, statins by 62%, and angiotensin-converting enzyme inhibitors or receptor antagonists by 61%. 75% were on nitrates, almost two-thirds on beta-blockers, and one-tenth on anticoagulants (Table 1).

### 3.2. Follow-Up

**Figure 1:**
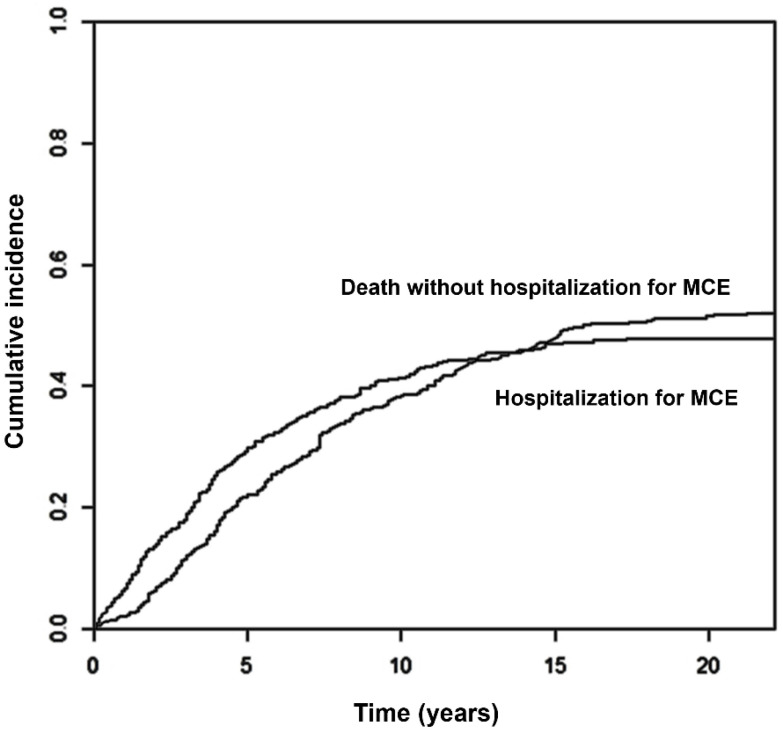
Cumulative incidence of admission for major cardiovascular event, with death without hospitalization as competing event. Abbreviations: MCE: major cardiovascular event.

**Figure 2:**
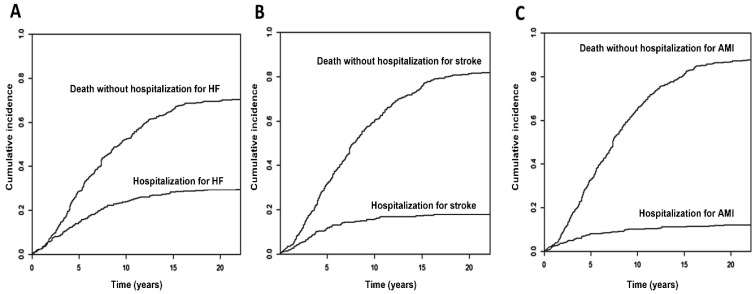
Cumulative incidence of hospital admission for each major cardiovascular event, with death without admission as a competing event. (**A**) Cumulative incidence of hospitalization for heart failure. (**B**) Cumulative incidence of hospitalization for stroke. (**C**) Cumulative incidence of hospitalization for acute myocardial infarction. Abbreviations: AMI: acute myocardial infarction; HF: heart failure.

After a follow-up period of up to 22 years (median 7 years, p25–p75 4–11 years), 198 patients (48%) experienced at least one MCE requiring hospitalization before their death. The most frequent event was HF, which occurred in 122 patients (209 hospitalizations); 74 patients presented with 81 strokes, and 49 patients suffered 58 AMIs. The cumulative incidences at 5, 10 and 20 years were 30% (95% Confidence Interval, 95% CI 25–34%), 41% (36–46%) and 48% (43–53%) for any event (Figure 1), 15% (11–18%), 24% (20–28%) and 30% (25–34%) for HF, 8% (5–11%), 10% (7–13%) and 12% (9–15%) for AMI and 12% (9–15%), 16% (12–19%) and 18% (14–22%) for stroke/TIA (Figure 2A–C).

### 3.3. Predictors of Major Cardiovascular Events. Univariate Analysis

A higher frequency of hospitalization for MCE was associated with a greater presence of hypertension, diabetes, HF, atrial fibrillation, cardiomegaly, and a higher prescription of angiotensin-converting enzyme inhibitors or angiotensin receptor antagonists, diuretics, digoxin, or anticoagulants, as well as a higher platelet count; and with lower haemoglobin levels, glomerular filtration rate, lower frequency of previous revascularization and treatment with antiplatelet drugs and statins (Table 1).

Variables associated with a higher frequency of HF hospitalizations were hypertension, diabetes, atrial fibrillation, previous HF, abnormal electrocardiogram, lower diastolic blood pressure values, glomerular filtration rate, and HDL cholesterol, higher triglyceride values, as well as lower prescription of antiplatelet and lipid-lowering therapy, and greater use of HF-related drugs (Table 2); in the case of AMI, only lower treatment with angiotensin-converting enzyme inhibitors or receptor antagonists was significant in the univariate analysis (Table 3). Finally, arterial hypertension, higher systolic blood pressure, and active smoking were significant predictors of stroke in the univariate analysis (Table 4). Full tables with univariate associations of baseline variables with components of the main end-point are available in Supplementary Material, Tables S1–S3.

### 3.4. Predictors of Major Cardiovascular Events. Multivariate Analysis

The independent multivariate predictors of hospitalization for MCE were hypertension (HR 1.58, [95% CI 1.15–2.18], *p* = 0.005), diabetes (HR 1.38 [1.03–1.85], *p* = 0.031), prior HF (HR 2.52 [1.59–4.01], *p* < 0.0005), and atrial fibrillation (HR 1.68 [1.13–2.50], *p* = 0.011). Of these, the presence of prior HF was the strongest predictor. Independent predictors of HF admission were arterial hypertension (HR 1.59 [1.04–2.44], *p* = 0.033), prior HF (HR 4.87 [2.87–8.29], *p* < 0.0005), AF (HR 2.41 [1.49–3.90], *p* < 0.0005), lower diastolic blood pressure (HR 0.98 [0.96–0.99], *p* = 0.022), and an abnormal EKG (HR 1.79 [1.15–2.78], *p* = 0.010). For stroke admission, hypertension (HR 2.19 [1.26–3.81], *p* = 0.006) and being an active smoker (HR 7.73 [1.83–32.61], *p* = 0.005) were found to be significant predictors. Multivariate analysis did not identify any different predictor than the only one variable that was significantly associated with AMI admission in the univariate analysis. Events per variable in the final multivariable models were 49.5, 24.4 and 37 for MCE, heart failure and stroke, respectively. Selecting initial variables to include in multivariate models with stricter criteria (*p* < 0.10) or repeating the analysis with a forward stepwise inclusion method yielded the same results. 

Taking into account the beta coefficients of the regression equation of multivariate model for predicting hospitalizations for MCE, an investigational score was assembled, assigning 1 point each to hypertension and diabetes mellitus, 2 points to atrial fibrillation and 3 points to prior HF (HA_2_D-HF_3_). Details on calculations and the performance of this score in our sample are described in Supplementary Material Table S4, Figures S1 and S2, with a C statistic of 0.61 (95% CI 0.56–0.67), *p* < 0.0005, and a 36% higher risk of MCE for each point (HR 1.36 [1.23–1.49], *p* < 0.0005). 

## 4. Discussion

The primary findings of our study were as follows: (a) there was a high incidence (48%) of MCE hospitalizations in a “real-world” cohort of patients aged ≥75 years with CCS over their lifetime; (b) the most frequent cause of hospitalization was HF; (c) arterial hypertension, diabetes mellitus, prior HF, and atrial fibrillation were independent predictors of MCE hospitalization in the multivariate analysis; and (d) other simple clinical variables also predict hospitalization for HF, stroke, and AMI. To the best of our knowledge, this is the first study to analyze the natural history of this elderly population with CCS over a 22-year period of follow-up.

### 4.1. Lifetime Incidence of Major Cardiovascular Events

Although other studies have examined the prognosis of CCS [2,18,19], the literature is scant on this subject in the older patient subgroup. With the exception of the TIME study [20] (mean age 80 ± 4 years), and more recently, a substudy of CORONOR [21] (age > 85 years), but with less than half the number of patients and a shorter follow-up time (4.7 years) than in our study. A substantial body of research on CCS patients has consistently documented a lower prevalence of non-fatal major events compared to the findings observed in our study. For instance, the CLARIFY registry [18] reported cumulative incidences of HF at 5 years of admission of 5.4%, non-fatal AMI of 2.8%, and stroke of 1.9%, which contrast with the 17%, 9%, and 14% observed in our study, respectively. In a separate study of patients over 85 years of age [21], the 10-year cumulative incidences of AMI and stroke were low (6.6% and 7.7%), compared to 14% and 22% in our series. However, the cumulative incidence of HF hospitalization was high (27.8%), although it was lower than in our series (34%), despite the older age of the patients in the study. In a subanalysis of patients aged ≥75 years in the ISCHEMIA study [22], the cumulative incidence of HF and stroke at 4 years was 4.7% and 3.3%, respectively. These figures are considerably lower than the 17% and 14% observed at 5 years in our study. However, a higher incidence of AMI was noted in the former study, with 13% of patients experiencing an event at 4 years compared to 9% at 5 years.

In our series, the most frequent cause of hospitalization was, by far, HF. This event might be globally underestimated, as other studies [23] indicate that the frequency of new-onset HF not requiring hospitalization can be up to four times higher. This finding is consistent with observations in national registries showing increasing incidences of HF in recent years among elderly patients [24]. Notably, the mean ejection fraction in our sample was preserved, with a value higher than that in other studies [23] analysing HF incidence in CCS patients. In those studies, a lower ejection fraction was a predictor of HF, which was not the case in our cohort, although their mean age was substantially lower. This predominance of preserved ejection fraction in this population of elderly CCS patients with high incidence of HF in follow up constitutes an appeal, in the contemporary management of these patients, to the widespread adoption of effective drugs for prevention of HF hospitalizations. In this sense, drugs as sodium-glucose cotransporter 2 inhibitors, which have demonstrated a clear reduction in HF events in patients with diabetes [25,26] and with HF with or without diabetes and independently of reduced or preserved ejection fraction [27,28,29,30], which were not available at the inclusion in our study, would have probably resulted in a significant impact in the reduction in events in a population similar to that of the present study.

### 4.2. Factors Related to the Incidence of MCE Hospitalization

Several factors may explain the high incidence of MCE in our study. First, the mean age was significantly higher than in many other CCS studies. Age is associated with both mortality and event rates in CCS [1,4,19,20]. Furthermore, our population included 36% women, whereas in younger samples [1,18,31] this percentage is typically 20–25%, consistent with the increasing incidence of CCS in women with advancing age [9].

Additionally, a high percentage of our patients had at least one prior ACS (90.6%) compared to studies such as BARIHD [19] or TIME [20]. Recent studies have shown that patients with a prior AMI have a higher risk of complications [18]. This is particularly relevant given our prior revascularization rate of 23.2%, compared to 40.7% in BARIHD [19], 82.1% in CLARIFY [18], and 96% in the REACH registry [32]. While the impact of revascularization on prognosis in CCS has shown beneficial effects in observational studies, it has failed to do so in randomized clinical trials [33,34,35]. However, percutaneous revascularization in ST-elevation ACS is the current standard of care, improving not only acute mortality but also late complications [36]. 

Our sample presented a high prevalence of hypertension, diabetes, and especially hypercholesterolemia, up to 72.8%, considerably higher than that of other CCS studies [18,19,20], although similar to studies with older populations [21]. 

Other differences with recent studies addressing younger populations lie in pharmacological treatment. For example, 63% of our patients received beta-blockers, a lower proportion than in CLARIFY [18] (75%) or REACH [32] (65%). In the BAHRID [19] study, beta-blocker treatment was a protective factor against MCE. Although recent evidence argues against the benefit of beta-blockers after AMI, this study includes a contemporary managed population with high rates of revascularization and optimal medical treatment, which is not comparable to our series [37]. The most notable differences, however, concern statin treatment, with a prescription rate of only 62%—much lower than in recent studies [18,19,21,32] (80–90%)—and a low proportion of high-potency statins. These lower rates of pharmacological treatment, consistent with the recruitment period in the early 2000s, could partially explain the higher MCE incidence compared to more recent studies. 

Furthermore, it should be noted that many of these studies utilized different definitions for MCE or analysed them as part of composite endpoints including overall or cardiovascular mortality. Consequently, comparisons must be interpreted with caution, and this could help to explain, at least in part, the discrepancies in event rates.

Finally, another factor to consider in the incidence of MCE hospitalization is the follow-up time. The median follow-up in our study was 7 years, longer than in studies such as BAHRID [19], TIME [20], or CORONOR [1] with 2, 3, and 2 years respectively, or the CORONOR substudy [21] in very elderly patients, which was 4.5 years. A longer follow-up allows for a more reliable characterization of the long-term incidence of cardiovascular events.

### 4.3. Independent Predictors of MCE Hospitalizations

Our study demonstrates that simple clinical variables can identify patients at higher risk of MCE hospitalization. These include arterial hypertension, diabetes mellitus, prior HF, and atrial fibrillation. While the role of hypertension as a risk factor for CHF is well established [7,38], its management in the elderly presents unique challenges. This population is characterized by a higher burden of comorbidities, which amplifies the risk of cardiovascular events, particularly heart failure. Furthermore, due to increased arterial stiffness, frailty, and isolated systolic hypertension, elderly patients often exhibit reduced tolerance to antihypertensive medication, frequently leading to orthostatic hypotension. This is particularly critical in patients with coronary artery disease, as an excessive reduction in diastolic pressure may compromise coronary perfusion, potentially precipitating myocardial ischemia [39]. Despite this, analyses derived from the SPRINT trial [40] and others [41] have shown that strict blood pressure control reduces the risk of AMI, stroke, HF, and cardiovascular death in older patients. 

Diabetes is a well-known predictor of mortality and MCE in CCS; in the BARIHD study [19], it was associated with cardiovascular death, and in CLARIFY [18], with the composite of death and non-fatal AMI. However, its role as a predictor of very long-term MCE in CCS patients is less well defined.

In our study, a history of HF emerged as the most powerful predictor of hospitalizations for MCE. This finding is consistent with prior evidence from younger CCS populations, such as the BAHRID study [19], where prior HF hospitalization was the strongest predictor of mortality, and the TIME [20] and CLARIFY [18] studies, where it independently predicted death or non-fatal AMI. Furthermore, in populations older than our cohort, prior HF has been identified as a strong predictor of mortality [21]; however, data regarding its impact on MCE hospitalizations in elderly CCS patients over such an extended follow-up period are lacking.

Finally, atrial fibrillation was identified as an independent predictor of MCE hospitalizations. Although it has been associated with the composite endpoint of death and non-fatal AMI in some studies [18], evidence remains scarce for CCS patients aged ≥75 years. The independent predictors for each specific MCE were notably similar to those for the composite endpoint, a finding likely driven by the predominance of HF, which had a 10-year cumulative incidence of 34% and accounted for more than half of all recorded events.

It is noteworthy the absence in the multivariate model of some protective medications, which were associated with fewer events in univariate analysis, as antiplatelets and statins. Maybe the identified predictors were simply more powerful, or perhaps, it indicated confounding by indication, where sicker patients were less likely to be prescribed these drugs. 

### 4.4. Strengths and Limitations

The main strength of our work lies in providing novel information in a patient subgroup for which little prior evidence exists, and with a very long-term follow-up, more extensive than any previously reported for this elderly population, with a large number of events, which allows us to understand their natural history until their death. Even accounting for death without events as a competing risk, a statistical strategy that avoids overestimation of risk [42], the high event rates found during follow-up highlight the particular fragility of this population within the spectrum of CCS. This can serve as an incentive to intensify pharmacological treatment and secondary prevention measures in these patients, with a special focus on addressing the very high risk and consequences of HF as the main event for these patients throughout their lives. Current evidence on the benefits of regular physical exercise, as well as new therapies for HF with preserved ejection fraction, as was the case for most patients in the series, could improve the prognosis in this population.

In turn, the identification of simple clinical predictors can help identify patients at higher risk of experiencing MCE admissions throughout their lives, thus allowing for more individualized management. The proposed HA_2_D-HF_3_ could be a useful tool for stratifying the risk of events and deciding a closer follow-up of these patients at higher risk, including perhaps biomarker surveillance of early signs of HF [43] even in specialized geriatric or HF units, which have demonstrated significant reduction in recurrent hospitalizations [44]. For those who present an HF event, another important measure would be enhancing transitional care management programs [45].

Our study has some limitations. First, it is a single-center study, which warrants caution in generalizing the results. Second, seven patients were excluded from the analysis (five due to loss to follow-up and two because they were still alive at the end of the follow-up period). This constitutes a potential limitation of the study. However, whether these patients had all been hospitalized for MCE or not, the difference would have been a cumulative incidence of 49% to 47%, which is of little clinical relevance.

Third, some baseline characteristics, such as obesity and peripheral arterial disease, were not fully recorded in the database and could not be analyzed; nor could other variables of recognized prognostic importance, such as depression, frailty, and the degree of isolation and social support. These unrecorded geriatric syndromes would have refined our ability to predict MCE and would be of outmost importance in personalizing treatment goals. We also could not track changes in medical treatment during evolution, a limitation inherent to a lifetime follow-up study. Furthermore, coronary anatomy was not known in all cases, so the proportion of patients with non-obstructive coronary artery disease could not be evaluated. We have proposed a new user-friendly HAD-HF_3_ score, but this tool needs an external validation in a contemporary managed population before widespread clinical application.

Finally, events were adjudicated manually by researchers, without evaluation by an external committee. Furthermore, since only events requiring hospital admission were recorded, we cannot ensure that there were no additional outpatient events. However, our interest was to evaluate hospitalizations, with the impact on quality of life and economic burden that they entail for patients and healthcare systems.

## 5. Conclusions

In this prospective, single-center study, almost half of this cohort of patients ≥75 years with CCS had at least one hospitalization for a major cardiovascular event during their lifetime. HF was the most frequent event. Various clinical variables such as prior HF, hypertension, diabetes mellitus, or the presence of atrial fibrillation could be useful for stratifying event risk.

## Figures and Tables

**Table 1:** General characteristics of the series and univariate predictors of hospitalization for major cardiovascular events.

Variable	Total N = 414	Major CV Events N = 198	No Major CV Events N = 216	Hazard Ratio (95% Confidence Interval)	*p*-Value
Demographics					
Age (years)	79.0 ± 3.7	79.0 ± 3.7	79.0 ± 3.6	1.02 (0.98–1.06)	0.248
Female Sex	149 (36%)	82 (41.4%)	57 (31%)	1.25 (0.94–1.60)	0.119
Risk factors					
**Arterial hypertension**	**265 (64.3%)**	**143 (72.6%)**	**122 (56.7%)**	**1.62 (1.18–2.22)**	**0.003**
**Diabetes mellitus**	**131 (31.8%)**	**74 (37.6%)**	**57 (26.5%)**	**1.46 (1.09–1.95)**	**0.011**
Dyslipidemia	268 (72.8%)	126 (72.8%)	142 (71.9%)	0.79 (0.57–1.11)	0.177
Active smoker	5 (1.2%)	2 (1.0%)	3 (1.4%)	2.18 (0.30–15.85)	0.440
Family history	17 (4.2%)	10 (5.2%)	7 (3.3%)	1.28 (0.68–2.43)	0.442
Prior disease					
Previous ACS	377 (91.3%)	183 (92.4%)	194 (90.2%)	1.37 (0.81–2.33)	0.237
Angina and ischemia	22 (5.3%)	8 (4.0%)	14 (6.5%)	0.60 (0.29–1.21)	0.152
Previous revascularization ^1^	108 (26.2%)	44 (22.2%)	64 (29.9%)	0.78 (0.55–1.08)	0.139
**Percutaneous**	**79 (19.1%)**	**29 (14.6%)**	**50 (23.3%)**	**0.66 (0.45–0.98)**	**0.040**
Surgical	35 (8.5%)	17 (8.6%)	18 (8.3%)	1.12 (0.68–1.84)	0.665
Previous stroke	17 (4.2%)	9 (4.6%)	8 (3.8%)	1.34 (0.68–2.63)	0.390
**Atrial fibrillation**	**50 (12.3%)**	**30 (15.3%)**	**20 (9.5%)**	**1.81 (1.23–2.68)**	**0.003**
**Heart failure**	**31 (7.5%)**	**22 (11.1%)**	**9 (4.2%)**	**2.86 (1.82–4.50)**	**<0.0005**
Symptoms and physical exam					
Angor FC ≥ II	99 (24%)	49 (24.9%)	50 (23.1%)	1.10 (0.79–1.51)	0.575
Baseline SBP (mmHg)	130 ± 17	131 ± 19	130 ± 16	1,00 (0.99–1.01)	0.514
Baseline DBP (mmHg)	73 ± 10	72 ± 10	73 ± 9	0.99 (0.97–1.00)	0.122
Baseline HR (lpm)	68 ± 11	69 ± 13	68 ± 10	1,01 (1.00–1.02)	0.240
Complementary tests					
Glucose (mg/dL)	117 ± 37	120 ± 44	114 ± 29	1.00 (1.00–1.01)	1.004
Total cholesterol (mg/dL)	193 ± 40	194 ± 41	192 ± 40	1.00 (1.00–1.01)	0.447
HDL cholesterol (mg/dL)	53 ± 14	53 ± 15	53 ± 13	1.00 (0.99–1.01)	0.786
LDL cholesterol (mg/dL)	116 ± 36	116 ± 36	115 ± 36	1.00 (1.00–1.01)	0.393
Triglycerides (mg/dL)	126 ± 79	127 ± 70	124 ± 86	1.00 (1.00–1.00)	0.480
**GFR (mg/min)**	**60.2 ± 15.0**	**54.3 ±14.6**	**65.6 ± 13.3**	**0.96 (0.94–0.98)**	**<0.0005**
**Hemoglobin (g/dL)**	**13.4 ± 1.5**	**13.1 ± 1.6**	**13.7 ± 1.3**	**0.82 (0.68–0.99)**	**0.039**
Leukocytes (10^3^/μL)	7.7 ± 2.1	7.8 ± 2.0	7.6 ± 2.1	1.12 (0.97–1.29)	0.124
**Platelets (10^3^/μL)**	**231.0 ± 76.2**	**236.4 ± 82.3**	**226.2 ± 70.6**	**1.04 (1.00–1.08)**	**0.043**
Abnormal ECG	231 (59.5%)	121 (64.4%)	110 (55%)	1.18 (0.87–1.58)	0.288
**Cardiomegaly**	**53 (15.1%)**	**32 (19.2%)**	**21 (11.5%)**	**2.04 (1.38–3.02)**	**<0.0005**
Baseline LVEF (%)	53.8 ± 14.6	54.4 ± 14.6	53.3 ± 14.6	1.00 (0.99–1.01)	0.960
Baseline treatment					
Antiplatelet therapy	353 (85.5%)	164 (82.8%)	189 (87.9%)	0.70 (0.48–1.01)	0.057
Aspirin	320 (77.5%)	151 (76.3%)	169 (78.6%)	0.76 (0.54–1.05)	0.098
**Vitamin K antagonists**	**46 (11.1%)**	**25 (12.6%)**	**21 (9.8%)**	**1.58 (1.03–2.40)**	**0.035**
Nitrates	307 (74.2%)	153 (77.3%)	154 (71.3%)	1.32 (0.94–1.84)	0.105
ACEI	207 (50.2%)	103 (52%)	104 (48.6%)	1.08 (0.81–1.42)	0.609
**ARB-II**	**50 (12.1%)**	**32 (16.2%)**	**18 (8.4%)**	**1.62 (1.11–2.37)**	**0.013**
ACEI/ARB-II	251 (60.9%)	130 (65.7%)	121 (56.5%)	1.33 (0.99–1.79)	0.056
Calcium channel blockers	201 (48.6%)	93 (47%)	108 (50%)	0.96 (0.73–1.27)	0.786
Beta-blockers	235 (63.2%)	107 (59.1%)	128 (67%)	0.86 (0.64–1.16)	0.338
**Statins**	**235 (62.2%)**	**105 (58.0%)**	**130 (66.0%)**	**0.72 (0.54–0.97)**	**0.032**
**Diuretics**	**181 (43.8%)**	**99 (50.0%)**	**82 (38.1%)**	**1.77 (1.34–2.35)**	**<0.0005**
**Digoxin**	**29 (7.0%)**	**19 (9.6%)**	**10 (4.7%)**	**2.20 (1.36–3.56)**	**0.001**

Data are expressed as mean ± standard deviation or number (valid percentage). ^1^ Revascularization was reported as percutaneous or surgical if any of these procedures had been performed in the patient, so the sum of both groups is higher than the total number or revascularized patients, as some patients had both types of revascularization performed. Abbreviations: ACEI, angiotensin-converting enzyme inhibitors; ACS: acute coronary syndrome; ARB-II: angiotensin II receptor antagonists. CV: cardiovascular. DBP: diastolic blood pressure; ECG: electrocardiogram; FC: functional class; GFR, glomerular filtration rate. HDL: high-density lipoprotein; HR: heart rate. LDL: low-density lipoprotein. LVEF, left ventricular ejection fraction. SBP: systolic blood pressure.

**Table 2:** Univariate predictors of a first hospitalization for heart failure (*p* < 0.15).

Variable	Heart Failure Hospitalization N = 122	No Hospitalization for Heart Failure N = 292	Hazard Ratio (95% Confidence Interval)	*p*-Value
**Arterial hypertension**	**88 (72.7%)**	**177 (60.8%)**	**1.66 (1.11–2.48)**	**0.013**
**Diabetes mellitus**	**49 (40.5%)**	**82 (28.2%)**	**1.74 (1.21–2.52)**	**0.003**
Active smoker	1 (0.8%)	4 (1.4%)	4.54 (0.61–33.63)	0.139
Previous ACS	115 (94.3%)	262 (90%)	1.85 (0.86–3.98)	0.114
**Angina and ischemia**	**1 (0.8%)**	**21 (7.2%)**	**0.11 (0.02–0.80)**	**0.029**
Previous revascularization ^1^	22 (18%)	86 (29.7%)	0.64 (0.40–1.02)	0.061
**Percutaneous**	**12 (9.8%)**	**67 (23%)**	**0.44 (0.24–0.80)**	**0.007**
**Atrial fibrillation**	**24 (19.8%)**	**26 (9.1%)**	**2.62 (1.67–4.11)**	**<0.0005**
**Heart failure**	**22 (18%)**	**9 (3.1%)**	**6.42 (3.96–10.40)**	**<0.0005**
**Baseline DBP (mmHg)**	**70.8 ± 9.8**	**73.6 ± 8.6**	**0.97 (0.95–0.99)**	**0.002**
Baseline HR (lpm)	69.0 ± 11.8	67.8 ± 11.2	1.01 (1.00–1.03)	0.102
**HDL cholesterol (mg/dL)**	**50.0 ± 13.0**	**54.1 ± 14.6**	**0.98 (0.96–1.00)**	**0.019**
**Triglycerides (mg/dL)**	**136.3 ± 79.5**	**121.3 ± 78.0**	**1.00 (1.00–1.01)**	**0.048**
**GFR (mg/min)**	**53.4 ±13.4**	**63.2 ± 14.8**	**0.96 (0.93–0.98)**	**0.001**
Platelets (10^3^/μL)	223.1 ± 90.0	230.1 ± 70.0	1.00 (1.00–1.01)	0.141
**Abnormal ECG**	**81 (71.7%)**	**150 (54.5%)**	**1.74 (1.15–2.63)**	**0.008**
**Cardiomegaly**	**26 (25.2%)**	**27 (10.9%)**	**3.27 (2.08–5.16)**	**<0.0005**
**Antiplatelet therapy**	**94 (77.0%)**	**259 (89.0%)**	**0.47 (0.31–0.72)**	**<0.0005**
Aspirin	92 (75.4%)	228 (78.4%)	0.71 (0.47–1.07)	0.099
**Vitamin K antagonists**	**22 (18.0%)**	**24 (8.2%)**	**2.66 (1.66–4.25)**	**<0.0005**
ACEI	70 (57.4%)	137 (47.2%)	1.42 (0.99–2.04)	0.056
**ARB-II**	**24 (19.7%)**	**26 (9.0%)**	**2.06 (1.32–3.22)**	**0.002**
**ACEI/ARB-II**	**92 (75.4%)**	**159 (54.8%)**	**2.32 (1.53–3.50)**	**<0.0005**
**Statins**	**57 (51.8%)**	**178 (66.4%)**	**0.58 (0.40–0.85)**	**0.005**
**Diuretics**	**68 (55.7%)**	**113 (38.8%)**	**2.31 (1.61–3.32)**	**<0.0005**
**Digoxin**	**17 (14.0%)**	**12 (4.1%)**	**3.61 (2.15–6.09)**	**<0.0005**

Data are expressed as mean ± standard deviation or number (valid percentage). ^1^ Revascularization was reported as percutaneous or surgical if any of these procedures had been performed in the patient, so the sum of both groups is higher than the total number or revascularized patients, as some patients had both types of revascularization performed. Abbreviations: ACEI, angiotensin-converting enzyme inhibitors; ACS: acute coronary syndrome; ARB-II: angiotensin II receptor antagonists. DBP: diastolic blood pressure ECG: electrocardiogram; GFR, glomerular filtration rate. HDL: high-density lipoprotein; HR: heart rate.

**Table 3:** Univariate predictors of a first hospitalization for acute myocardial infarction (*p* < 0.15).

Variable	Hospitalization for Acute Myocardial Infarction N = 50	No Hospitalization for Acute Myocardial Infarction N = 364	Hazard Ratio (95% Confidence Interval)	*p*-Value
Arterial hypertension	27 (54%)	238 (65.7%)	0.65 (0.37–1.13)	0.125
Active smoker	1 (2.0%)	4 (1.1%)	5.27 (0.71–39.16)	0.105
Angor FC ≥ II	16 (32.0%)	83 (22.9%)	1.57 (0.86–2.84)	0.139
Baseline DBP (mmHg)	75.3 ± 8.6	72.5 ± 9.1	1.03 (1.00–1.06)	0.094
Total cholesterol (mg/dL)	201.4 ± 42.9	191.4 ± 39.9	1.01 (1.00–1.01)	0.117
LDL cholesterol (mg/dL)	124.0 ± 36.5	114.5 ± 36.1	1.01 (1.00–1.02)	0.094
ACEI	20 (40.0%)	187 (51.7%)	0.65 (0.37–1.14)	0.136
**ACEI/ARB-II**	**23 (46.0%)**	**228 (63%)**	**0.55 (0.32–0.97)**	**0.038**
Calcium channel blockers	31 (62.0%)	170 (46.7%)	1.67 (0.94–2.96)	0.079
Beta-blockers	25 (52.1%)	210 (64.8%)	0.64 (0.37–1.13)	0.127

Data are expressed as mean ± standard deviation or number (valid percentage). Abbreviations: ACEI, angiotensin-converting enzyme inhibitors; ARB-II: angiotensin II receptor antagonists. FC: functional class; LDL: low-density lipoprotein.

**Table 4:** Univariate predictors of a first hospitalization for stroke (*p* < 0.15).

Variable	Hospitalization for Stroke N = 74	No Hospitalization for Stroke N = 340	Hazard Ratio (95% Confidence Interval)	*p*-Value
**Arterial hypertension**	**58 (78.4%)**	**207 (61.2%)**	**2.15 (1.24–3.75)**	**0.007**
Diabetes mellitus	28 (37.8%)	103 (30.5%)	1.45 (0.90–2.32)	0.125
**Active smoker**	**2 (2.7%)**	**3 (0.9%)**	**7.00 (1.66–29.47)**	**0.008**
Previous revascularization ^1^	14 (18.9%)	94 (27.8%)	0.65 (0.36–1.16)	0.141
Previous stroke	5 (6.8%)	12 (3.6%)	2.11 (0.84–5.26)	0.110
**Baseline SBP (mmHg)**	**134.7 ± 18.2**	**129.2 ± 16.9**	**1.02 (1.00–1.03)**	**0.013**
GFR (mg/min)	55.9 ± 13.0	61.2 ± 15.3	0.97 (0.94–1.01)	0.096

Data are expressed as mean ± standard deviation or number (valid percentage). ^1^ Revascularization was reported as percutaneous or surgical if any of these procedures had been performed in the patient, so the sum of both groups is higher than the total number of revascularized patients, as some patients had both types of revascularization performed. Abbreviations: GFR, glomerular filtration rate. SBP: systolic blood pressure.

## Data Availability

The data supporting the findings of this study will not be freely available due to sensitivity reasons but can be provided upon reasonable request to the corresponding author.

## Supplementary Materials

The following supporting information can be downloaded at: https://www.mdpi.com/article/10.3390/jcm15010207/s1.
