# Supplementary material for: Hospitalizations for Major Cardiovascular Events in Patients Aged 75 Years or Older with Chronic Coronary Syndrome for the Whole Life Span
Source: J Clin Med. 2025 Dec 27;15(1):207. doi: 10.3390/jcm15010207 (PMC12786816; doi:10.3390/jcm15010207)
Supplement: Supplementary file 1 [file jcm-15-00207-s001.zip › jcm-4028533-supplementary.pdf]

Supplementary Material

Hospitalizations for major cardiovascular events in patients aged 75 years or older with chronic coronary syndrome for the whole life span.

Life-long CV morbidity in elderly coronary patients

Lucas Barreiro Mesa [1], Martín Ruiz Ortiz [1,2,3,4*], Josué Lopez Baizán [1] , Leticia Mateos de la Haba [1], Cristina Ogayar Luque [1], José Javier Sánchez Fernández [1], Elías Romo Peña [1], Mónica Delgado Ortega [1,2,3], Ana Rodríguez Almodovar [1,2,3], Fátima Esteban Martinez [1], Manuel Anguita Sanchez [1,2,3], Rafael González Manzanares [1,2,3], Juan Carlos Castillo Domínguez [1,2,3], José López Aguilera [1,2,3], Amador López Granados [1], Manuel Pan Álvarez-Ossorio [1,2,3] and Dolores Mesa Rubio [1,2,3].

1. Cardiology Department, Hospital Universitario Reina Sofía, University of Córdoba, Córdoba, Spain
2. Maimónides Biomedical Research Institute of Córdoba (IMIBIC), University of Córdoba, Córdoba, Spain
3. Cardiovascular Diseases Biomedical Research Networking Centre (CIBERCV), Carlos III Health Institute, Madrid, Spain
4. Faculty of Health Sciences, Isabel I International University of Castile, Burgos, Spain

* Correspondence: author: Martín Ruiz Ortiz. e-mail: maruor@gmail.com. Telephone number: +34 626 93 58 78 ORCID ID 0000-0002-1479-7044

Index

Table S1. Univariate association of baseline variables with heart failure hospitalization in follow-up…………………………………………………………………………..3

Table S2. Univariate association of baseline variables with acute myocardial infarction hospitalization in follow-up …………………………………………………………..…5

Table S3. Univariate association of baseline variables with stroke hospitalization in follow-up ………………………...…….…………………………………...…………7

Table S4. Calculation of score points for HA_2D-HF_3 score.……..……………………...9

Figure S1. Distribution and events of HA_2D-HF_3 score in our sample.………...…..….10

Figure S2. ROC curve analysis for HA_2D-HF_3 score in our sample………...…………11

Table S1. Univariate association of baseline variables with heart failure hospitalization in follow-up

Variable	Heart failure hospitalization N= 122	No hospitalization for heart failure N= 292	Hazard Ratio (95%Confidence Interval)	P-value
Age (years)	78.8 ± 3.6	79.1 ± 3.7	1.02 (0.97-1.07)	0.538
Female Sex	52 (42.6%)	97 (33.2%)	1.30 (0.91-1.86)	0.154
Arterial hypertension	88 (72.7%)	177 (60.8%)	1.66 (1.11-2.48)	0.013
Diabetes mellitus	49 (40.5%)	82 (28.2%)	1.74 (1.21-2.52)	0.003
Dyslipidemia	77 (72.6%)	191 (72.9%)	0.78 (0.51-1.21)	0.269
Active smoker	1 (0.8%)	4 (1.4%)	4.54 (0.61-33.63)	0.139
Family history	7 (5.9%)	10 (3.5%)	1.73 (0.80-3.71)	0.161
Previous ACS	115 (94.3%)	262 (90%)	1.85 (0.86-3.98)	0.114
Angina and ischemia	1 (0.8%)	21 (7.2%)	0.11 (0.02-0.80)	0.029
Previous revascularization[1]	22 (18%)	86 (29.7%)	0.64 (0.40-1.02)	0.061
Percutaneous	12 (9.8%)	67 (23%)	0.44 (0.24-0.80)	0.007
Surgical	10 (8.2%)	25 (8.6%)	1.19 (0.62-2.27)	0.603
Previous stroke	4 (3.3%)	13 (4.5%)	0.93 (0.34-2.53)	0.884
Atrial fibrillation	24 (19.8%)	26 (9.1%)	2.62 (1.67-4.11)	0.000
Heart failure	22 (18%)	9 (3.1%)	6.42 (3.96-10.40)	0.000
Angor FC≥ II	30 (24.8%)	69 (23.6%)	1.10 (0.73-1.67)	0.640
Baseline SBP (mmHg)	128.7 ± 19.1	130.8 ± 16.4	1.00 (0.98-1.01)	0.373
Baseline DBP (mmHg)	70.8 ± 9.8	73.6 ± 8.6	0.97 (0.95-0.99)	0.002
Baseline HR (bpm)	69.0 ± 11.8	67.8 ± 11.2	1.01 (1.00-1.03)	0.102
Glucose (mg/dl)	116.6 ± 34.0	117.4 ± 38.2	1.00 (0.99-1.01)	0.741
Total cholesterol (mg/dl)	191.5 ± 42.6	193.1 ± 39.4	1.00 (1.00-1.01)	0.919
HDL cholesterol (mg/dl)	50.0 ± 13.0	54.1 ± 14.6	0.98 (0.96-1.00)	0.019
LDL cholesterol (mg/dl)	114.5 ± 37.3	116.0 ± 35.8	1.00 (0.99-1.01)	0.964
Triglycerides (mg/dl)	136.3 ± 79.5	121.3 ± 78.0	1.00 (1.00-1.01)	0.048
GFR (mg/min)	53.4 ±13.4	63.2 ± 14.8	0.96 (0.93-0.98)	0.001
Hemoglobin (g/dl)	13.4 ±1.6	13.5 ± 1.5	0.93 (0.74-1.19)	0.577
Leukocytes (10^3/µL)	7.6 ± 2.2	7.7 ±2.0	1.07 (0.88-1.29)	0.506
Platelets (10^3/µL)	223.1 ± 90.0	230.1 ± 70.0	1.00 (1.00-1.01)	0.141
Abnormal ECG	81 (71.7%)	150 (54.5%)	1.74 (1.15-2.63)	0.008
Cardiomegaly	26 (25.2%)	27 (10.9%)	3.27 (2.08-5.16)	<0.0005
Baseline LVEF (%)	53.5 ±15.3	53.9 ±14.3	1.00 (0.98-1.01)	0.490
Antiplatelet therapy	94 (77.0%)	259 (89.0%)	0.47 (0.31-0.72)	<0.0005
Aspirin	92 (75.4%)	228 (78.4%)	0.71 (0.47-1.07)	0.099

Vitamin K antagonists	22 (18.0%)	24 (8.2%)	2.66 (1.66-4.25)	0.000
Nitrates	92 (75.4%)	215 (73.6%)	1.14 (0.75-1.72)	0.541
ACEI	70 (57.4%)	137 (47.2%)	1.42 (0.99-2.04)	0.056
ARB-II	24 (19.7%)	26 (9.0%)	2.06 (1.32-3.22)	0.002
ACEI/ARB-II	92 (75.4%)	159 (54.8%)	2.32 (1.53-3.50)	<0.0005
Calcium channel blockers	51 (41.8%)	150 (51.4%)	0.78 (0.54-1.12)	0.177
Beta-blockers	64 (56.1%)	171 (66.3%)	0.76 (0.53-1.11)	0.155
Statins	57 (51.8%)	178 (66.4%)	0.58 (0.40-0.85)	0.005
Diuretics	68 (55.7%)	113 (38.8%)	2.31 (1.61-3.32)	<0.0005
Digoxin	17 (14.0%)	12 (4.1%)	3.61 (2.15-6.09)	<0.0005

Data are expressed as mean±standard deviation or number (valid percentage). [1]Revascularization was reported as percutaneous or surgical if any of these procedures had been performed in the patient, so the sum of both groups is higher than the total number or revascularized patients, as some patients had both types of revascularization performed. Abbreviations: ACEI, angiotensin-converting enzyme inhibitors; ACS: acute coronary syndrome; ARB-II: angiotensin II receptor antagonists. CV: cardiovascular. DBP: diastolic blood pressure ECG: electrocardiogram; FC: functional class; GFR, glomerular filtration rate. HDL: high-density lipoprotein; HR: heart rate. LDL: low-density lipoprotein. LVEF, left ventricular ejection fraction. SBP: systolic blood pressure. TGL: triglycerides.

Table S2. Univariate association of baseline variables with acute myocardial infarction hospitalization in follow-up

Variable	Hospitalization for acute myocardial infarction N= 50	No hospitalization for acute myocardial infarction N= 364	Hazard Ratio (95%Confidence interval)	P-value
Age (years)	78.5 ± 3.5	79.1 ± 3.7	1.00 (0.92-1.08)	0.903
Female Sex	14 (28%)	135 (37.1%)	0.64 (0.34-1.18)	0.152
Arterial hypertension	27 (54%)	238 (65.7%)	0.65 (0.37-1.13)	0.125
Diabetes mellitus	16 (32%)	115 (31.8%)	1.09 (0.60-1.97)	0.784
Dyslipidemia	35 (81.4%)	233 (71.7%)	1.37 (0.63-2.97)	0.421
Active smoker	1 (2.0%)	4 (1.1%)	5.27 (0.71-39.16)	0.105
Family history	3 (6.1%)	14 (4.0%)	1.69 (0.52-5.46)	0.379
Previous ACS	45 (90.0%)	332 (91.5%)	1.04 (0.41-2.61)	0.942
Angina and ischemia	4 (8.0%)	18 (4.9%)	0.21 (0.43-3.39)	0.719
Previous revascularization[1]	12 (24.0%)	96 (26.5%)	0.89 (0.47-1.71)	0.733
Percutaneous	7 (14.0%)	72 (19.8%)	0.66 (0.30-1.47)	0.311
Surgical	5 (10.0%)	30 (8.2%)	1.37 (0.54-3.45)	0.510
Previous stroke	1 (2.0%)	16 (4.5%)	0.55 (0.08-3.99)	0.553
Atrial fibrillation	2 (4.1%)	48 (13.4%)	0.38 (0.09-1.56)	0.178
Heart failure	3 (6.0%)	28 (7.7%)	1.24 (0.38-4.00)	0.724
Angor FC≥ II	16 (32.0%)	83 (22.9%)	1.57 (0.86-2.84)	0.139
Baseline SBP (mmHg)	129.9 ± 13.6	130.2 ± 17.7	1.00 (0.98-1.02)	0.879
Baseline DBP (mmHg)	75.3 ± 8.6	72.5 ± 9.1	1.03 (1.00-1.06)	0.094
Baseline HR (bpm)	66.3 ± 14.2	68.4 ± 10.9	0.99 (0.96-1.02)	0.355
Glucose (mg/dl)	125.8 ± 52.3	115.8 ± 32.8	1.01 (1.00-1.02)	0.230
Total cholesterol (mg/dl)	201.4 ± 42.9	191.4 ± 39.9	1.01 (1.00-1.01)	0.117
HDL cholesterol (mg/dl)	55.0 ± 12.7	52.6 ± 14.4	1.01 (0.99-1.03)	0.482
LDL cholesterol (mg/dl)	124.0 ± 36.5	114.5 ± 36.1	1.01 (1.00-1.02)	0.094
Triglycerides (mg/dl)	127.3 ± 71.8	125.5 ± 79.7	1.00 (1.00-1.01)	0.692
GFR (mg/min)	57.8± 15.6	60.5 ± 15.0	0.99 (0.95-1.03)	0.522
Hemoglobin (g/dl)	13.2 ± 1.6	13.5 ± 1.5	0.83 (0.58-1.20)	0.324
Leukocytes (10^3/μL)	7.8 ± 1.5	7.7 ± 2.2	1.12 (0.87-1.45)	0.383
Platelets (10^3/μL)	223.7 ± 43.2	232.1 ± 80.3	1.00 (0.99-1.01)	0.815
Abnormal ECG	30 (61.2%)	201 (59.3%)	1.08 (0.61-1.93)	0.787
Cardiomegaly	6 (14.3%)	47 (15.3%)	1.30 (0.54-3.10)	0.559
Baseline LVEF (%)	53.7 ± 15.3	53.8 ± 14.5	1.00 (0.97-1.02)	0.713

Antiplatelet therapy	42 (84.0%)	311 (85.7%)	0.73 (0.34-1.56)	0.410
Aspirin	38 (76.0%)	282 (77.7%)	0.71 (0.37-1.38)	0.318
Vitamin K antagonists	2 (4.0%)	44 (12.1%)	0.44 (0.11-1.82)	0.257
Nitrates	39 (78.0%)	268 (73.6%)	1.27 (0.65-2.49)	0.485
ACEI	20 (40.0%)	187 (51.7%)	0.65 (0.37-1.14)	0.136
ARB-II	5 (10.0%)	45 (12.4%)	0.80 (0.32-2.02)	0.641
ACEI/ARB-II	23 (46.0%)	228 (63%)	0.55 (0.32-0.97)	0.038
Calcium channel blockers	31 (62.0%)	170 (46.7%)	1.67 (0.94-2.96)	0.079
Beta-blockers	25 (52.1%)	210 (64.8%)	0.64 (0.37-1.13)	0.127
Statins	29 (58.0%)	206 (62.8%)	0.76 (0.43-1.34)	0.352
Diuretics	18 (36.0%)	163 (44.9%)	0.90 (0.50-1.62)	0.733
Digoxin	2 (4.0%)	27 (7.5%)	0.71 (0.17-2.94)	0.636

Data are expressed as mean±standard deviation or number (valid percentage). [1]Revascularization was reported as percutaneous or surgical if any of these procedures had been performed in the patient, so the sum of both groups is higher than the total number or revascularized patients, as some patients had both types of revascularization performed. Abbreviations: ACEI, angiotensin-converting enzyme inhibitors; ACS: acute coronary syndrome; ARB-II: angiotensin II receptor antagonists. CV: cardiovascular. DBP: diastolic blood pressure ECG: electrocardiogram; FC: functional class; GFR, glomerular filtration rate. HDL: high-density lipoprotein; HR: heart rate. LDL: low-density lipoprotein. LVEF, left ventricular ejection fraction. SBP: systolic blood pressure. TGL: triglycerides.

Table S3. Univariate association of baseline variables with stroke hospitalization in follow-up

Variable	Hospitalization for stroke N= 74	No hospitalization for stroke N= 340	Hazard Ratio (95%Confidence interval)	P-value
Age (years)	79.3 ± 4.0	78.9 ± 3.6	1.04 (0.98-1.11)	0.161
Female Sex	30 (40.5%)	119 (35.0%)	1.18 (0.74-1.88)	0.476
Arterial hypertension	58 (78.4%)	207 (61.2%)	2.15 (1.24-3.75)	0.007
Diabetes mellitus	28 (37.8%)	103 (30.5%)	1.45 (0.90-2.32)	0.125
Dyslipidemia	45 (71.4%)	223 (73.1%)	0.73 (0.42-1.26)	0.262
Active smoker	2 (2.7%)	3 (0.9%)	7.00 (1.66-29.47)	0.008
Family history	5 (6.8%)	12 (3.6%)	1.73 (0.70-4.30)	0.237
Previous ACS	66 (89.2%)	311 (91.7%)	0.93 (0.45-1.94)	0.846
Angina and ischemia	6 (8.1%)	16 (4.7%)	1.37 (0.59-3.15)	0.464
Previous revascularization[1]	14 (18.9%)	94 (27.8%)	0.65 (0.36-1.16)	0.141
Percutaneous	11 (14.9%)	68 (20.1%)	0.70 (0.37-1.34)	0.285
Surgical	5 (6.8%)	30 (8.8%)	0.85 (0.34-2.10)	0.723
Previous stroke	5 (6.8%)	12 (3.6%)	2.11 (0.84-5.26)	0.110
Atrial fibrillation	10 (13.5%)	40 (12%)	1.36 (0.70-2.65)	0.370
Heart failure	5 (6.8%)	26 (7.6%)	1.31 (0.52-3.26)	0.566
Angor FC$\geq$ II	16 (21.6%)	83 (24.5%)	0.91 (0.52-1.58)	0.731
Baseline SBP (mmHg)	134.7 ± 18.2	129.2 ± 16.9	1.02 (1.00-1.03)	0.013
Baseline DBP (mmHg)	72.6 ± 8.6	72.8 ± 9.2	0.99 (0.97-1.02)	0.603
Baseline HR (bpm)	68.8 ± 12.7	68.0 ± 11.1	1.01 (0.99-1.03)	0.329
Glucose (mg/dl)	120.1 ± 40.5	116.5 ± 36.1	1.00 (0.99-1.02)	0.473
Total cholesterol (mg/dl)	193.7 ± 35.2	192.4 ± 41.4	1.00 (1.00-1.01)	0.703
HDL cholesterol (mg/dl)	55.2 ± 18.5	52.4 ± 13.1	1.01 (0.99-1.03)	0.229
LDL cholesterol (mg/dl)	117.3 ± 30.9	115.2 ± 37.3	1.00 (1.00-1.01)	0.538
Triglycerides (mg/dl)	119.6 ± 58.4	127.0 ± 82.4	1.00 (0.99-1.00)	0.691
GFR (mg/min)	55.9 ± 13.0	61.2 ± 15.3	0.97 (0.94-1.01)	0.096
Hemoglobin (g/dl)	13.1 ± 1.5	13.5 ± 1.5	0.85 (0.63-1.14)	0.268
Leukocytes (10^3/µL)	8.0 ± 2.1	7.6 ±2.1	1.13 (0.92-1.40)	0.248
Platelets (10^3/µL)	237.8 ± 70.6	229.4 ± 77.7	1.00 (1.00-1.01)	0.281
Abnormal ECG	42 (59.2%)	189 (59.6%)	0.92 (0.57-1.48)	0.728
Cardiomegaly	9 (14.3%)	44 (15.3%)	1.29 (0.63-2.62)	0.488
Baseline LVEF (%)	56.3 ±13.5	53.3 ±14.8	1.01 (0.99-1.03)	0.352

Antiplatelet therapy	66 (89.2%)	287 (84.7%)	1.28 (0.62-2.67)	0.508
Aspirin	57 (77.0%)	263 (77.6%)	0.83 (0.48-1.42)	0.488
Vitamin K antagonists	5 (6.8%)	41 (12.1%)	0.72 (0.29-1.78)	0.472
Nitrates	55 (74.3%)	252 (74.1%)	1.03 (0.61-1.74)	0.909
ACEI	34 (45.9%)	173 (51.2%)	0.83 (0.52-1.31)	0.422
ARB-II	11 (14.9%)	39 (11.5%)	1.35 (0.71-2.57)	0.356
ACEI/ARB-II	43 (58.1%)	208 (61.5%)	0.93 (0.59-1.48)	0.762
Calcium channel blockers	39 (52.7%)	162 (47.6%)	1.24 (0.78-1.95)	0.364
Beta-blockers	38 (60.3%)	197 (63.8%)	0.92 (0.55-1.52)	0.735
Statins	37 (56.9%)	198 (63.3%)	0.72 (0.44-1.17)	0.185
Diuretics	33 (44.6%)	148 (43.7%)	1.26 (0.79-1.99)	0.330
Digoxin	3 (4.1%)	26 (7.7%)	0.66 (0.21-2.10)	0.481

Data are expressed as mean±standard deviation or number (valid percentage). [1]Revascularization was reported as percutaneous or surgical if any of these procedures had been performed in the patient, so the sum of both groups is higher than the total number or revascularized patients, as some patients had both types of revascularization performed. Abbreviations: ACEI, angiotensin-converting enzyme inhibitors; ACS: acute coronary syndrome; ARB-II: angiotensin II receptor antagonists. CV: cardiovascular. DBP: diastolic blood pressure ECG: electrocardiogram; FC: functional class; GFR, glomerular filtration rate. HDL: high-density lipoprotein; HR: heart rate. LDL: low-density lipoprotein. LVEF, left ventricular ejection fraction. SBP: systolic blood pressure. TGL: triglycerides.

Table S4. Calculation of score points for HA_2D-HF_3 score

Variable	Hazard Ratio	Beta coefficient	Crude point estimate	Rounded point estimate
Hypertension	1.58	0.46	1.44	1
Atrial fibrillation	1.68	0.52	1.63	2
Diabetes	1.38	0.32	1	1
Heart Failure	2.52	0.92	2.88	3

Calculation of point estimates was performed according to the method described by Rassi et al. Each regression coefficient of variables independently associated to major cardiovascular event hospitalizations in the final multivariate Cox model was divided by the smallest coefficient and rounded to the nearest integer.

Figure S1. Distribution and events of HA_2D-HF_3 score in our sample

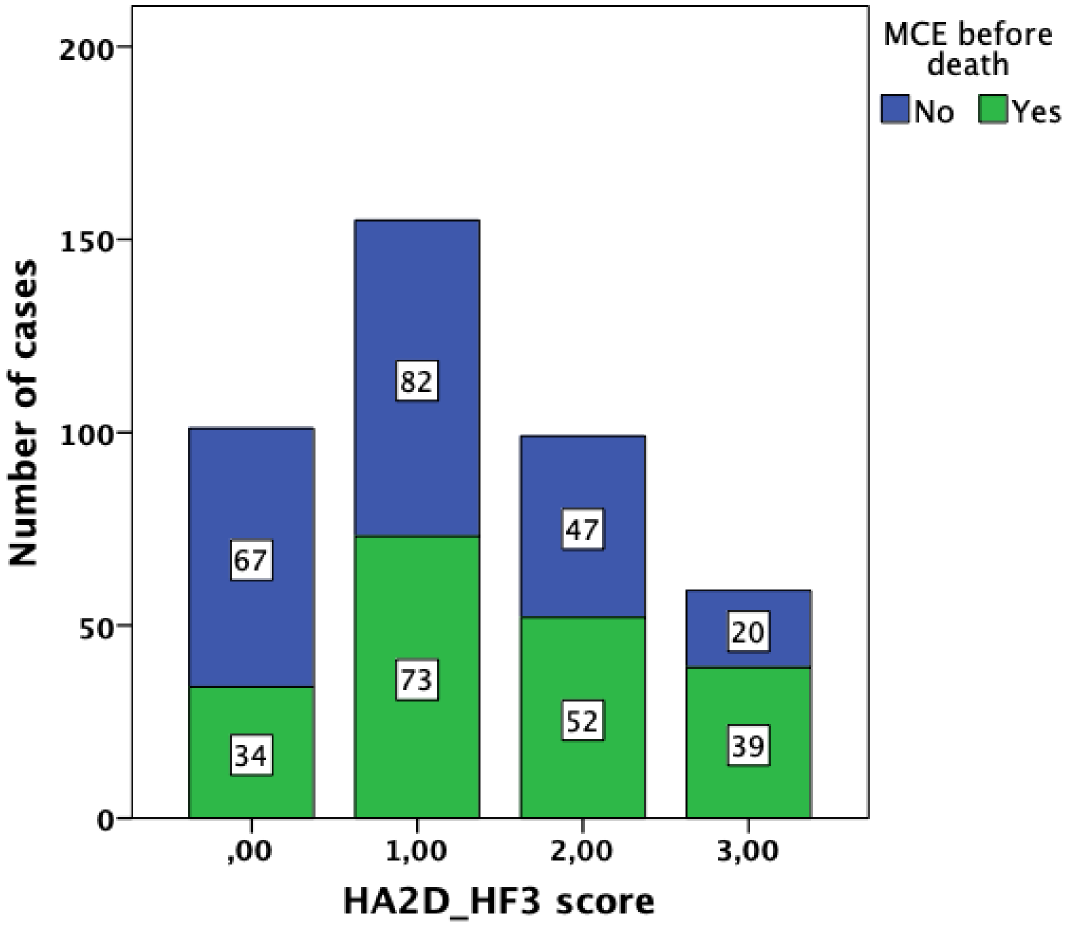

Abbreviations: HA_2D-HF_3 score: hypertension (1 point), atrial fibrillation (2 points), diabetes (1 point), heart failure (3 points); MCE: major cardiac events.

Figure S2. ROC curve analysis for HA_2D-HF_3 score in our sample

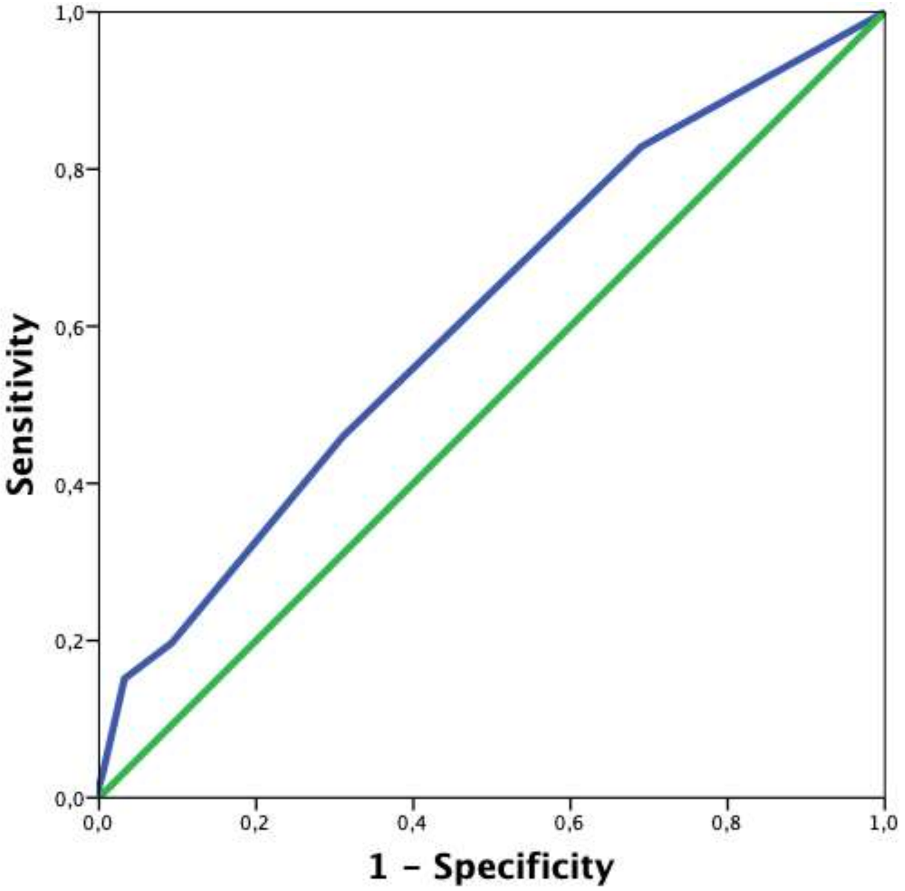

Area under curve (C statistics) 0.61 (95% confidence interval 0.56-0.67), p<0.0005
